# Food load manipulation ability shapes flight morphology in females of central-place foraging Hymenoptera

**DOI:** 10.1186/1742-9994-10-36

**Published:** 2013-06-28

**Authors:** Carlo Polidori, Angelica Crottini, Lidia Della Venezia, Jesús Selfa, Nicola Saino, Diego Rubolini

**Affiliations:** 1 Departamento de Biodiversidad y Biología Evolutiva, Museo Nacional de Ciencias Naturales (CSIC), C/ José Gutiérrez Abascal 2, 28006 Madrid, Spain; 2 CIBIO, Centro de Investigação em Biodiversidade e Recursos Genéticos, Campus Agrário de Vairão, Rua Padre Armando Quintas, Vairão, Vila do Conde 4485-661, Portugal; 3 Department of Biology, McGill University, Stewart Biology Building, Docteur Penfield 1205, Montreal, Quebec H3A 1B1, Canada; 4 Departament de Zoologia, Universitat de València, C/Dr. Moliner 50, València, Burjassot 46100, Spain; 5 Dipartimento di Bioscienze, Università degli Studi di Milano, Via Celoria 26, Milano 20133, Italy

**Keywords:** Bees, Flight Muscle Ratio, Foraging, Wasps, Wing Loading

## Abstract

**Background:**

Ecological constraints related to foraging are expected to affect the evolution of morphological traits relevant to food capture, manipulation and transport. Females of central-place foraging Hymenoptera vary in their food load manipulation ability. Bees and social wasps modulate the amount of food taken per foraging trip (in terms of e.g. number of pollen grains or parts of prey), while solitary wasps carry exclusively entire prey items. We hypothesized that the foraging constraints acting on females of the latter species, imposed by the upper limit to the load size they are able to transport in flight, should promote the evolution of a greater load-lifting capacity and manoeuvrability, specifically in terms of greater flight muscle to body mass ratio and lower wing loading.

**Results:**

Our comparative study of 28 species confirms that, accounting for shared ancestry, female flight muscle ratio was significantly higher and wing loading lower in species taking entire prey compared to those that are able to modulate load size. Body mass had no effect on flight muscle ratio, though it strongly and negatively co-varied with wing loading. Across species, flight muscle ratio and wing loading were negatively correlated, suggesting coevolution of these traits.

**Conclusions:**

Natural selection has led to the coevolution of resource load manipulation ability and morphological traits affecting flying ability with additional loads in females of central-place foraging Hymenoptera. Release from load-carrying constraints related to foraging, which took place with the evolution of food load manipulation ability, has selected against the maintenance of a powerful flight apparatus. This could be the case since investment in flight muscles may have to be traded against other life-history traits, such as reproductive investment.

## Introduction

Flying animals show a huge diversity of body shapes and structures and, as a consequence, a great variation in flight performance that in turn largely affects the ability to avoid predators, chase mates and carry food items [1-4]. An intriguing question is thus whether variation in flight morphology is adaptively tuned to specific ecological conditions, such as habitat type, or to specific behavioural traits, such as food preferences [5]. Flying animals must generate a lift force sufficient to counteract the gravitational force acting on their bodies, and this requirement is frequently exacerbated when an additional load has to be carried in flight, which commonly occurs during foraging [6,7]. Load-lifting and manoeuvrability limits may constrain foraging and entail important ecological and evolutionary consequences.

Based on previous theoretical and empirical studies it is possible to make predictions about the relationship between morphology, lift production, power output and take-off ability. Experiments on insects, birds and bats have revealed that flight muscle ratio (i.e., the flight muscle mass to body mass ratio, FMR) is the most important determinant of take-off ability with additional loads [6]. Because flying animals generate an approximately constant force per unit of flight muscle during high-intensity bursts of flight [6], FMR also affects, together with other morphological traits (e.g. position of center of body mass), acceleration and, partly, manoeuvrability in flight (since it could be considered as a series of changes in acceleration) [1,2]. Furthermore, relatively larger wings compared to body size, corresponding to a lower wing loading (WL) (i.e., the body mass to wing area ratio) are also associated with a superior flying ability [2,8]. Animals with lower WL can perform a more energetically efficient flight [9,10] and take off at higher speed (though cruising flight speed increases with WL [11]).

Studies of insects have shown that intraspecific variation in flight morphology also has important fitness consequences: for example, higher FMR in males has been related to a better competitive ability in territorial wasps [12] and dragonflies [1,13]. In a pompilid wasp, competitively successful males are larger, with a tendency for reduced WL [3]. Comparative studies are scarce, but still suggest the same patterns. For example, ant-attended aphid species have higher WL and smaller amount of flight muscles (implying a lower dispersal ability) than non-ant-attended species [14,15]. Similarly, palatable and non-mimetic butterflies have higher FMR, enhancing their escape ability [2]. Interestingly, in both aphids and butterflies, species with reduced flight thorax muscle mass allocate more resources to reproduction (e.g. ovarian size) [2,14,15], suggesting a trade-off between investment in flight muscles and reproduction.

On the whole, these studies indicate that flight morphology in insects is shaped by multiple, potentially contrasting selection pressures, including the ability to defend a territory and the ability to escape from predators, though other sources of selection have not yet been investigated. In particular, despite the known role of foraging behaviour and diet type in shaping the flight morphology of predatory vertebrates [8,16], to the best of our knowledge no study has examined these evolutionary relationships among predatory insects.

Flying, central-place foraging Hymenoptera (Aculeata), whose females repeatedly return with a food load to their nest in order to provision their immature brood, are an excellent system to study the evolutionary relationships between foraging ecology and flight morphology. This diverse group includes bees and wasps and shows huge variation in foraging ecology, as it includes predators hunting arthropods or other animal sources as well as pollen/nectar foragers [17]. Importantly, central-place foraging aculeates vary markedly in their foraging mode: some species, such as bees and social vespid wasps, have evolved the ability to modify the shape and size of food material and thereby the load carried in flight before transporting it to the nest. Bees can tune pollen and nectar load, whereas social vespid wasps often divide large prey in pieces and carry only parts of them on each foraging trip [18,19] (here defined as “able to manipulate” species, AtM species hereafter). In contrast, solitary vespid wasps and apoid wasps can only hunt and carry entire prey items to provision their brood. These species (here defined as “unable to manipulate” species, UtM species hereafter) should thus select prey weighing less than or equal to the maximum load they can carry in flight [7]. Because wasps able to divide in pieces large prey can successfully return by flying to the nest with food [20], while wasps unable to do this, in the same condition, often fail to forage (though in certain species females would shift to prey dragging over the ground [7]), we hypothesized that inability to modulate load size, rather than the type of food consumed (prey or pollen), should impose foraging constraints to UtM species, and that such constraints should lead to the evolution of a flight morphology that maximizes the ability to carry heavier loads.

UtM species are predicted to have therefore evolved a higher FMR and a lower WL, unless investment in flight muscles and wing size is counteracted by contrasting selection on other life-history traits, such as reproductive investment. Here we tested the prediction that variation in FMR and WL is associated with food load manipulation ability in central-place foraging Hymenoptera, with UtM species expected to have higher FMR and lower WL than AtM species. Furthermore, across species, we also expected these traits relevant to flight performance to have coevolved, with species having high FMR also having a lower WL.

## Results

**Figure 1:**
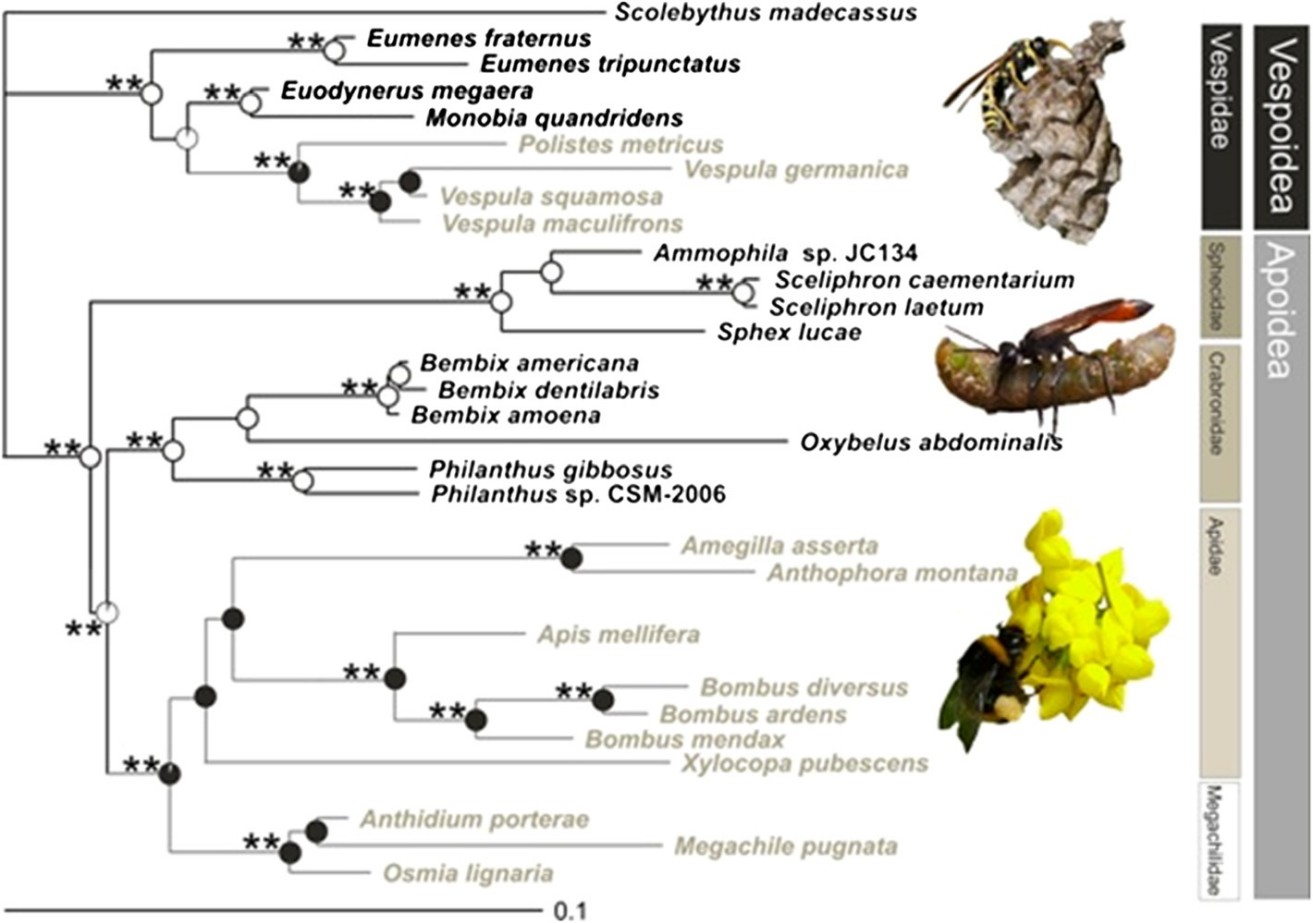
**Partitioned Bayesian tree based on a 50% majority rule consensus tree from the analysis of 1433 bp of the 18S rRNA and 28S rRNA gene fragments sequences of the selected Hymenoptera taxa. ***Scolebythus madecassus* was used as an outgroup. Asterisks denote Bayesian posterior probabilities values: *, 95–98%; **, 99–100%. Maximum Likelihood Markov model (Mk1) ancestral state reconstruction describing the food load manipulation ability on the MrBayes topology: AtM (“able to manipulate” species, names in grey) vs. UtM (“unable to manipulate” species, names in black). Pie diagrams at each node indicate the proportion of the Maximum Likelihood supporting alternative reconstructed character states. Bars define Families and Superfamilies. Species names refer to the sample used to build the phylogenetic tree; for correspondence with the morphologically studied species, see Additional file 1: Table S2. Pictures show, from top to down, *Polistes* sp. (Vespidae) at nest, *Ammophila* sp. (Sphecidae) with prey and *Bombus* sp. foraging on flowers (Apidae).

The identification of the ancestral state on the reconstructed phylogeny revealed that food manipulation ability is a derived trait for both Apoidea and Vespoidea, with the more primitive species all being unable to manipulate load size (Figure 1). Furthermore, character mapping revealed that food manipulation ability has independently evolved twice in this set of Hymenoptera species, once in Apoidea and the other in Vespoidea (Figure 1).

**Figure 2:**
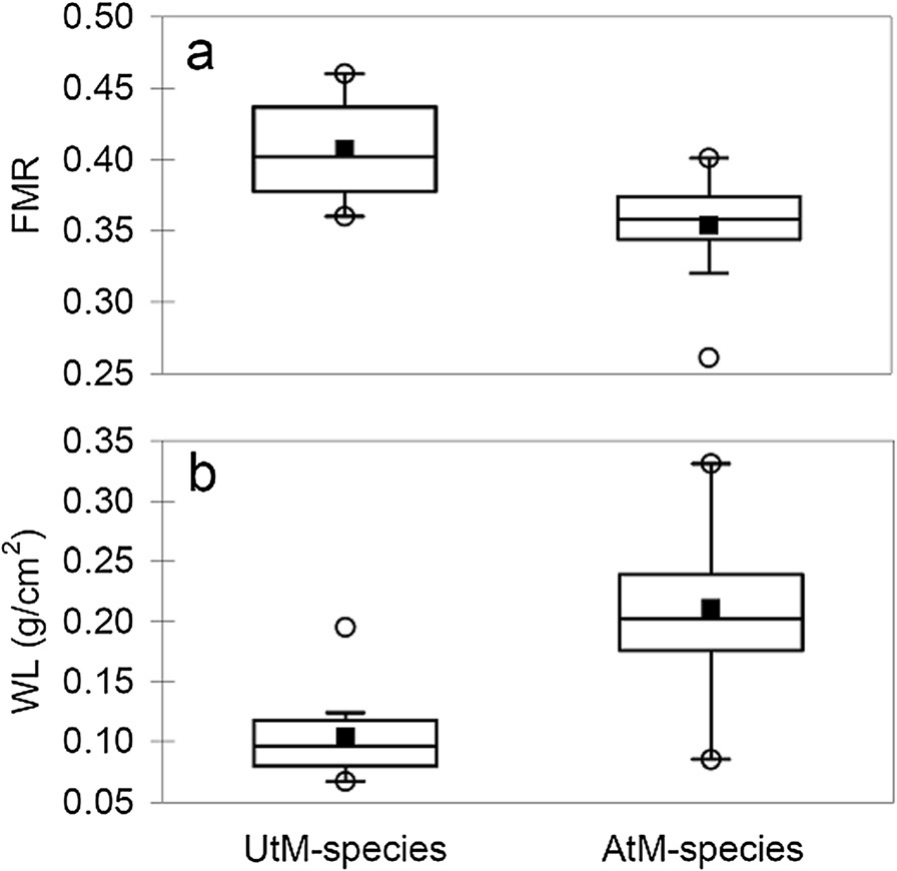
**Box-and-whisker plots of flight muscle ratio and wing loading in relation to food load manipulation ability. a** flight muscle ratio (FMR). **b** wing loading (WL). Medians (horizontal lines within boxes), means (■), 1° and 3° quartile (top and bottom horizontal lines of the boxes), as well as maximum and minimum values (○) are shown for the species able to manipulate the food load (AtM-species) and for the species unable to manipulate the food load (UtM). Endpoints of the whiskers represent the lowest datum still within 1.5 × interquartile range of the lower quartile, and the highest datum still within 1.5 × interquartile range of the upper quartile.

Our species’ sample encompassed a huge range of body masses (M_b_), from very small species weighing < 0.01 g to very large ones weighing 0.85 g (Table 1). After controlling for phylogeny and body mass, food load manipulation significantly predicted FMR (Table 2). Specifically, AtM-species had smaller FMR than UtM ones (Table 1 and Table 2, Figure 2). Body mass did not covary with FMR, and the estimated value of λ indicated that FMR showed almost no phylogenetic dependence (LR test, P = 0.99) (Table 2), suggesting that phylogenetic constraints did not affect the evolution of FMR in this species’ set.

**Table 1:** **Dataset used for comparative analyses**, **including wet body mass** (**M**_**b**_), **flight muscle ratio** (**FMR**), **wing loading** (**WL**), **dietary specialization** (**pollen**/**nectar or animal protein**) **and food manipulation ability** (**0** = **unable to manipulate food load**, **1** = **able to manipulate food load**)

**Taxonomy**	**Species**	**Species used in the phylogenetic reconstruction**	**Diet**	**Food manipulation ability**	**M**_**b **_**(g)**	**FMR**	**WL (g/cm**^**2**^**)**	**Source**
Apoidea: Apidae	*Amegilla dawsoni*	*Amegilla asserta*	Pollen	1	0.700	-	0.31	[21]
Apoidea: Apidae	*Anthophora* sp.	*Anthophora montana*	Pollen	1	0.133	0.396	0.183	This study
Apoidea: Apidae	*Apis mellifera*	*Apis mellifera*	Pollen	1	0.094	0.358	0.170	This study
Apoidea: Apidae	*Bombus impatiens*	*Bombus diversus*	Pollen	1	0.201	0.261	0.287	[22]
Apoidea: Apidae	*Bombus* sp. 1	*Bombus ardens*	Pollen	1	0.208	0.374	0.220	This study
Apoidea: Apidae	*Bombus* sp. 2	*Bombus mendax*	Pollen	1	0.204	0.401	0.199	This study
Apoidea: Apidae	*Xylocopa varipuncta*	*Xylocopa pubescens*	Pollen	1	0.838	0.342	0.331	[23]
Apoidea: Megachilidae	*Anthidium manicatum*	*Anthidium porterae*	Pollen	1	0.154	0.344	0.205	This study
Apoidea: Megachilidae	*Megachile rotundata*	*Megachile pugnata*	Pollen	1	0.102	0.32	0.178	This study
Apoidea: Megachilidae	*Osmia rufa*	*Osmia lignaria*	Pollen	1	0.187	0.353	0.223	This study
Apoidea: Sphecidae	*Ammophila sabulosa*	*Ammophila sp*. JC134	Prey	0	0.026	0.409	0.075	This study
Apoidea: Sphecidae	*Sceliphron curvatum*	*Sceliphron caementarium*	Prey	0	0.083	0.46	0.094	This study
Apoidea: Sphecidae	*Sceliphron destillatorium*	*Sceliphron laetum*	Prey	0	0.181	0.44	0.124	This study
Apoidea: Sphecidae	*Sphex rufocinctus*	*Sphex lucae*	Prey	0	0.118	0.426	0.109	This study
Apoidea: Crabronidae	*Bembix olivacea*	*Bembix americana*	Prey	0	0.109	0.46	0.122	This study
Apoidea: Crabronidae	*Bembix sinuata*	*Bembix dentilabris*	Prey	0	0.158	0.456	0.195	This study
Apoidea: Crabronidae	*Bembix troglodytes*	*Bembix amoena*	Prey	0	0.099	0.36	-	[24]
Apoidea: Crabronidae	*Oxybelus* sp.	*Oxybelus abdominalis*	Prey	0	0.008	0.374	0.087	This study
Apoidea: Crabronidae	*Philanthus pulchellus*	*Philanthus gibbosus*	Prey	0	0.043	0.392	0.08	This study
Apoidea: Crabronidae	*Philanthus triangulum*	*Philanthus sp*. CSM-2006	Prey	0	0.092	0.401	0.116	This study
Vespoidea: Vespidae	*Polistes dominulus*	*Polistes metricus*	Prey	1	0.065	0.369	0.085	This study
Vespoidea: Vespidae	*Vespula germanica*	*Vespula germanica*	Prey	1	0.067	0.361	-	[20]
Vespoidea: Vespidae	*Vespula maculifrons*	*Vespula maculifrons*	Prey	1	0.038	0.381	-	[20]
Vespoidea: Vespidae	*Vespula vulgaris*	*Vespula squamosa*	Prey	1	0.078	0.344	0.139	This study
Vespoidea: Vespidae	*Eumenes* sp. 1	*Eumenes fraternus*	Prey	0	0.048	0.403	0.078	This study
Vespoidea: Vespidae	*Eumenes* sp. 2	*Eumenes tripunctatus*	Prey	0	0.042	0.375	0.098	This study
Vespoidea: Vespidae	*Euodynerus* sp.	*Euodynerus megaera*	Prey	0	0.049	0.363	0.067	This study
Vespoidea: Vespidae	*Monobia quandridens*	*Monobia quandridens*	Prey	0	0.218	0.385	-	[25]

**Table 2:** **PGLS models testing the effect of food load manipulation ability** (**0** = **UtM**; **1** = **AtM**) **on flight muscle ratio** (**FMR**) **and** (**log**_**10**_-**transformed**) **wing**-**loading** (**WL**), **while controlling for wet body mass** (**log**_**10**_-**transformed**)

**Model**	**Estimate (s.e.)**	**t**	**P**	**λ**
*FMR* (*n* = *27 species*)				
Food load manipulation ability	-0.059 (0.016)	-3.71	0.001	0.01
Body mass	0.013 (0.021)	0.64	0.53	
*WL* (*n* = *24 species*)				
Food load manipulation ability	0.148 (0.059)	2.49	0.021	0.70
Body mass	0.278 (0.053)	5.25	< 0.001	

The maximum likelihood estimate value of λ, assessing the degree of phylogenetic dependence among the tested variables (see Materials and methods), is shown for each model.

The maximum food load that could be theoretically carried in flight by a species (Load_max_) was estimated to range from 0.007 g to over 0.7 g (0.14 ± 0.01 g on average) (Additional file 1: Table S2). The total load that could be lifted (M_max_ = Load_max_ + M_b_) ranged from 0.015 g to 1.55 g. In turn, UtM-species were predicted to carry loads weighing 118 ± 0.06% of their body mass, while this value was reduced to 86 ± 0.06% of body mass in AtM-species (Additional file 1: Table S2).

Wing size (A_w_) was also very variable among species, with values ranging from less than 0.1 cm^2^ to > 2 cm^2^ (Additional file 1: Table S2). WL was smaller in UtM-species compared to AtM ones, after controlling for phylogeny and body mass (Table 2, Figure 2). In this model, body mass strongly positively covaried with WL (Table 2) (see also Materials and methods), and the degree of phylogenetic dependence was relatively large (0.70, Table 2) and statistically significant (LR test, P = 0.011), Thus, differently from FMR, WL showed a high degree of phylogenetic autocorrelation, with closely related species showing more similar WL values than distantly related ones.

Results concerning the effect of food manipulation ability on FMR and WL were qualitatively unaltered if we used FMR and WL values based on dry mass (see Materials and methods) and head width instead of body mass as a covariate, despite the smaller sample of 21 species (Additional file 1: Table S3).

Since in our database all UtM species were arthropod predators, and most AtM species were pollen/nectar feeders (the exception were the few social vespid wasps), the strict association between dietary specialization (pollen/nectar vs. animal proteins) and food load manipulation ability may have confounded the above results. We therefore conducted an additional analysis of the effect of food manipulation ability on flight traits by excluding all the pollen/nectar feeders (i.e., bees) and restricting the dataset to wasps (including both UtM species, n = 14, and AtM species, n = 4). The analysis was conducted only on FMR, since sample size for WL was too small (only 2 AtM species). Despite the small sample size, the results confirmed the previous analyses, with significantly smaller FMR in the few AtM (0.364 ± 0.008) compared to the UtM species (0.407 ± 0.01) (PGLS model accounting for heterogeneity of variances and body mass; effect of food manipulation ability: -0.036 ± 0.017, t_15_ = 2.15, P = 0.048, λ = 0.26; further details not shown for brevity).

**Figure 3:**
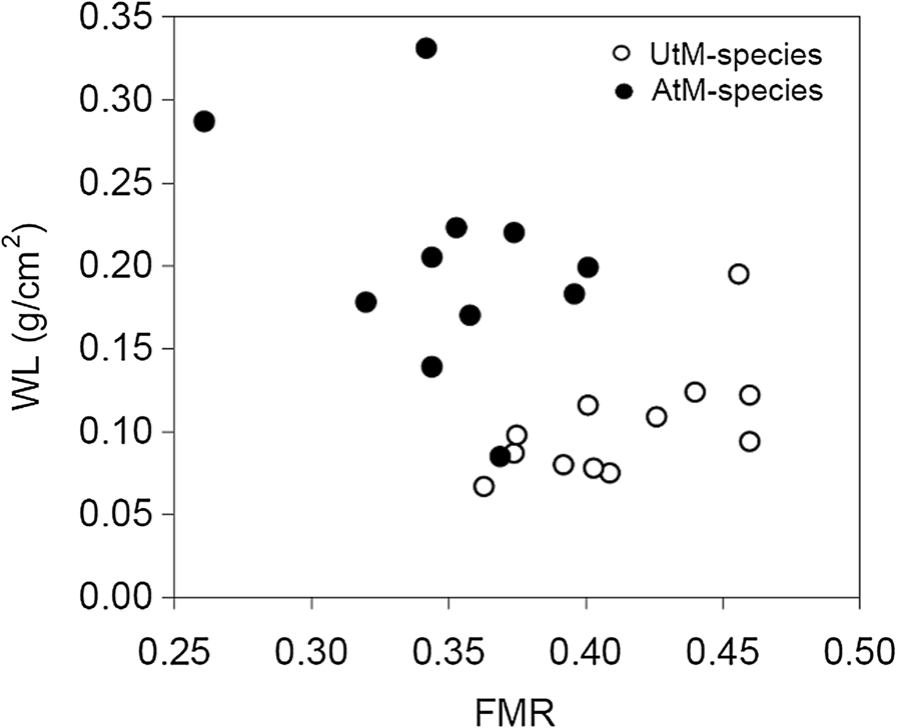
**Relationship between of WL and FMR.** The correlation is based on a total of 23 species, i.e. those for which both variables were available.

The covariation between WL and FMR across species was negative and statistically significant (PGLS model accounting for body mass, FMR estimate: -1.27 ± 0.42, t_20_ = -3.03, P = 0.007, λ = 0.82; body mass estimate 0.34 ± 0.05, t_20_ = 6.43, P < 0.001) (Figure 3).

## Discussion

We showed that the evolution of food load manipulation ability, which has occurred independently twice in the present set of species, is associated with a decrease of FMR and an increase in WL across species. Moreover, a high FMR was associated with a low WL, indicating coevolution of morphological traits related to foraging.

Within, as well as across, wasp species, the dependence of the maximum theoretical load that could be carried (Load_max_) on body mass allows larger individuals/species to potentially carry large prey, even though there is no relationship between FMR and body mass (Table 2). Empirical studies of individual wasp species indeed showed that larger individuals carry larger prey compared to smaller ones [20,25,26]. Moreover, larger species appear to be able to carry heavier loads (Pearson correlation test, r = 0.95, P < 0.001, data from 12 species reviewed by [7]). However, the degree to which individuals and species maximize their food load is highly variable, suggesting that other factors, such as prey specialization [27], prey availability [28] and intra-specific competition [24] may affect the food load mass. For example, females of *Bembix troglodytes*, a solitary wasp of about 0.01 g, hunt for flies weighing only half the theoretical maximum they can carry [24]. This may be a strategy to limit the attack of conspecific kleptoparasitic females, since carrying small prey may allow entering nest holes more rapidly and avoiding harassment [24]. On the other hand, the species-specialist cicada-hunting wasp *Sphecius convallis* carries prey loads approaching the theoretical maximum value of Load_max_, possibly because the strong specialization on a single prey species has allowed selection to adjust the morphology of females to an almost ideal size [27].

As re-calculated from data provided in a recent review [7], UtM-species carry on average loads weighing 89 ± 14% of body mass (data from 11 species of Sphecidae, Crabronidae and Vespidae), similar to the predicted % Load_max_/M_b_ average value of 118% on the entire set of species considered in this study (89.3% with data for the shared UtM-species in our study and [7], n = 7 species). Moreover, among UtM species, there was no correlation between prey size and FMR (Pearson correlation test, r = 0.43, P = 0.19, data from 11 species reviewed by [7]). It is believed that the first apoid wasps were specialized in hunting large orthopterans, and the prey spectrum would have then become broader to include smaller arthropods as diverse as flies, bees, beetles and spiders [29]. Thus, the evolution of flight morphology adapted to carry large items possibly preceded, during evolution, prey diversification, and did not change too much thereafter in apoid wasps. A change in flight morphology may have appeared when bees (*sensu stricto*, i.e. pollen/nectar foragers) separated from apoid wasps about 140 to 110 m.y.a. [30]. A similar pattern may have occurred among Vespidae, where solitary species (subfamily Eumeninae), unable to manipulate food load size, are basal to the social species [31], which are able to manipulate food. In Eumeninae, prey diversification was less pronounced, with only lepidopteran or coleopteran larvae used as prey, and a change in flight morphology may have appeared when eusociality, together with its associated foraging mode (e.g. direct liquid-feeding from adult foragers to larvae, made possible because of food manipulation ability) evolved [32].

The maintenance of higher FMR in UtM-species could also depend on the fact that the position of load during carrying may affect the center of gravity, unbalancing the wasp while flying. A recently developed model shows that, among species carrying prey impaled on the sting (i.e. well posterior to the wasp center of gravity), the Load_max_ can be severely reduced compared to the expected value [33]. Wasps can limit to some extent this problem by increasing the angle which the straight line connecting the wasp with the load center of mass makes with the horizontal line [33], but, evolutionarily speaking, any increase in FMR, positively affecting Load_max_, would help in carrying larger prey in such an unbalanced flight mode.

On the other hand, AtM species are far from approaching the average values of relative load size observed among UtM species, as they appear carrying loads weighing only 31 ± 6% (calculated from data of 17 species provided in [20,34-36]). This value is much lower than the average % Load_max_ estimated for our set of AtM-species (86%).

Actually, females of UtM-species were sometimes observed to return to the nests with a prey weighing more than Load_max_, implying an unsuccessful take-off [7]. With very large prey items, UtM wasps can potentially shift to an alternative strategy, such as carrying the prey to the nest by dragging it on the ground. Such behaviour has been described in some (but not all) species, though flight transportation was the preferred option if prey size is adequate [7]. For example, *Ammophila* spp. typically drag large caterpillar prey over the ground, but shift to flight transportation in case of smaller prey (reviewed in [37]). Indeed, dragging a prey on the ground may make it much more vulnerable to kleptoparasites and predators. The cicada-hunting wasp *Sphecius speciosus* can drag very large cicadas over a distance sometimes full of obstacles (e.g. dense and high grass) which makes prey prone to be abandoned and exploited by ants [7]. Dragging a prey over such complex substrates may also increase the duration of the hunting trip compared to flying across the same distance. Despite hunting site-nest distances are not provided and conclusions cannot be really drawn, data from the literature reported very short hunting trips (≤ 2 minutes) apparently only for flight-carrying wasps (e.g. [38-41]). Thus, although in specific cases wasps can use the alternative strategy of dragging very large prey, we expect a fitness advantage in term of foraging efficiency (e.g. number of prey hunted per day) when the prey is carried in flight. Clearly, a robust and direct comparison of the actual fitness costs and benefits of prey carrying in flight vs. prey dragging would be needed to confirm this speculation.

An interesting consequence of load-lifting/manoeuvrability constraints concerns diet composition and resource specialization. In bees, any individual could have access to its preferred resource (assuming these are available in the foraging environment) because food collection is only limited by the number of pollen grains it can carry and by the nectar volume it can ingest, i.e. by volumetric, not mass, constraints. In wasps that are able to manipulate food load the situation is similar: Coelho and Hoagland [20] studied the foraging behaviour of *Vespula germanica* on dead honeybees, and found that foragers too small to carry entire honeybees simply chopped body parts and took off with a smaller portion [20], without the need to discard the food item and search for a new, smaller one. Food manipulation ability would thus help to exploit the target food in a highly efficient way. At the same time, AtM-wasps can have access to a wider range of prey types, since also large prey, once chopped, can be readily exploited. Such increased efficiency in foraging may have even been important in promoting the evolution of eusociality, since social behaviour is unstable unless it provides important economic benefits and fitness gains to the individuals [42]. As a matter of fact, eusociality arose at least five times independently within AtM-lineages, while apparently only once within UtM-lineages (see also [43,44] for theoretical predictions on the link between food resource and social evolution in Hymenoptera).

On the other side, wasp species unable to manipulate food load will face a more adverse situation if the prey item is too large to be carried, and the wasp has to spend additional time and energy to search for a different, smaller, profitable prey [7]. In a simple model, Polidori et al. [45] predicted that wasp species hunting for hemimetabolous prey can be so affected by the body growth of their preferred prey during the course of the breeding season that they may be forced to shift to different prey species at a certain point. Later, a study on the orthopteran-hunting wasp, *Stizus continuus*, confirmed this prediction [46]. Furthermore, this shift to smaller prey species is confined in such wasps to only few other species, given their phylogenetic constraint in prey selection (typically prey species belong to one single order [17]), so that overall prey spectrum cannot be wide as in AtM-wasps.

As an additional advantage, higher FMR would enhance the escape ability of UtM-species, given that FMR is correlated with linear acceleration and the ability to accelerate vertically against gravity [2] and with flight speed [47]. Low WL is expected to confer similar advantages. In UtM-wasps, which are also those limited to hunt for living prey, lower WL could increase manoeuvrability during prey transportation, but could also increase efficiency while pursuing a living prey (e.g. via reduced minimum flight-speed requirement and turning radius), including fast-flying insects [48]. A similar example involves bats, in which species with greater WL forage in areas where there are fewer obstacles to detect and avoid [49]. On the other side, increased WL consistently decreased escape performance in a bird [50].

Despite higher WL requires higher wing-beat frequency and increases flight cost [51], flight speed generally increases with WL [11,52], so that higher WL could be positively selected in specific contexts. For example, males of perching butterfly species (which sit and wait on prominent landmarks and rapidly take off to intercept females) had higher WL than patrolling closely-related species [53]. AtM-species may thus fly faster while sacrificing manoeuvrability (an important factor while carrying loads) than UtM-species. These considerations, together with the observed negative covariation of FMR and WL across species, suggest that the selection pressures related to foraging (load-lifting/manoeuvrability) constitute the main determinants of flight morphology in central-place foraging Hymenoptera.

Moreover, among AtM species, investment in flight muscles and wing size, which would be of limited adaptive value during foraging, may further be counteracted by contrasting selection acting on other life-history traits, such as reproductive investment. In butterflies, for example, palatable species have higher FMR because of higher predation risk and stronger escape demands, but also smaller ovaries than unpalatable species, suggesting that investment in the flight apparatus and predator avoidance trades off with investment in reproduction [2]. Intriguingly, a preliminary analysis based on literature data suggests that this could be the case also among the Apoidea. In fact, the number of ovarioles per ovary was significantly higher in bees (3.6 ± 0.09, n = 32 species) than in apoid wasps (2.9 ± 0.03, n = 75 species) (t_39_ = 6.8, P < 0.0001) (data from [54,55]; highly eusocial bee species (queens), parasitoid apoid wasps and brood-parasitic apoid wasps were not considered because of their peculiar life-style). Though it might be speculated that eusocial bee species represent an exception to this pattern, we note that honeybee workers can have from 1 to 12 ovarioles per ovary (with an average of about 4) [56], thus roughly agreeing with the rest of bees. In addition, workers of one species of *Bombus* (the primitively eusocial *B*. *morio*) have the same number of ovarioles per ovary as related solitary species (4) [54]. This hypothesis, however, needs a robust, phylogenetically controlled, test, using species for which both flight morphology and measurements of fecundity are available.

## Conclusion

Our findings suggest that load-carrying constraints related to foraging have affected the evolution of flight morphology in flying central-place foraging Hymenoptera, and that release from these constraints, which took place with the evolution of food load manipulation ability, has selected against the maintenance of a costly flight apparatus, which could possibly be traded against reproductive investment.

## Materials and methods

### Sample collection and morphological measurements

We collected data on species from the two superfamilies Apoidea (families Apidae, Crabronidae, Megachilidae, Sphecidae) and Vespoidea (family Vespidae), including bees (pollen/nectar collectors) and wasps (prey collectors) (Table 1). A total of 216 females from 21 species [5–35 females per species, 10.8 ± 7.2 (s.d.) females per species on average] were caught in the field, in natural populations found in the Parque Natural de la Albufera, Valencia province (South-Eastern Spain), during the spring-summer 2009–2010. Specimens were determined to species level following taxonomic keys [57-59] and with the aid of experts; however, for seven of them we could only reach the genus level and therefore assigned specimens to morphospecies (Table 1). Additional data for seven species were obtained from the literature (Table 1). Overall, 14 species fell in the AtM group and 14 species in the UtM group. Bees and wasps were killed by freezing upon collection. Within 2–3 hours, females were weighed in the lab with an electronic balance (to the nearest 0.002 g) (M_b_, body mass). We then separated the thorax from the rest of the body and weighed it (M_t_, thorax mass). FMR was calculated as (0.95 × M_t_/M_b_) for individual specimens [6], and the average value within species was used in interspecific comparisons. For each species, we further calculated the predicted maximum food load mass which can be carried in flight after a successful take-off (Load_max_) according to the regression equation of maximum lift force vs. flight muscle mass for bees and wasps provided in Table five of [6]. Finally, by adding M_b_ to Load_max_, we calculated the total maximum load mass which can be carried (M_max_).

One wing pair (forewing and hindwing) was gently separated from the thorax, and then scanned on an Epson 2450 flatbed scanner (720 dpi). NIH ImageJ was used to determine individual wing area; total wing area (A_w_) refers to the area of both wing pairs and was obtained by doubling the previous measurements. We then calculated the wing loading WL (M_b_/A_w_) [10] for each individual, and the average value within species was used in interspecific comparisons. Measures were taken to the nearest 0.002 mm.

As wet body mass can be confounded by body condition and water content, we repeated the calculations of FMR and WL using dry mass (after oven-drying all body parts for 48 hours at 70 °C). In addition, we also measured the head width (a good predictor of body size in Hymenoptera, e.g. [60]) with a digital calliper (to the nearest 0.02 mm) to obtain a condition-independent body size estimate (Additional file 1: Table S2). For the sample of species we collected, the correlation between wet and dry mass was very high (r = 0.89, n = 21 species, log_10_-transformed variables). Moreover, there was a strict positive correlation between head width and wet mass (r = 0.90, n = 21 species, log_10_-transformed variables). Therefore, in the following analysis we used wet mass instead of dry mass because dry mass was not available for the seven species for which we obtained data from the literature (Table 1). Using wet mass instead of dry mass did not affect our conclusions (see also Results), as wet body mass truly reflects across-species differences in body size. Importantly, it is the wet mass that needs to be lifted by the insects and therefore it is the most relevant variable to measure from an eco-evolutionary standpoint.

For the morphological variables we recorded (M_b_, M_t_, A_w_), the variance among species was significantly larger than the variance within species (*F*-values always > 18, P < 0.0001).

### Molecular analyses

In comparative studies, species cannot be considered as independent sampling units as their shared ancestry may affect actual phenotypic values [61,62]. For this reason, we built a molecular phylogeny of the studied species to conduct comparative analyses accounting for phylogenetic relationships among species.

Tissue samples of the measured individuals could not be stored in suitable conditions to allow for genetic analyses; we therefore used sequences of a fragment of the 18S rRNA gene and of the 28S rRNA gene of 28 selected taxa of Apoidea and Vespoidea retrieved from GenBank. Whenever possible, we retrieved sequences from the same species for which we had morphological data; if no sequences were available for a given species, sequences from congeneric species were used. The complete list of taxa and GenBank accession numbers is provided in Additional file 1: Table S1. Homologous 18s rRNA and 28s rRNA gene sequences of *Scolebythus madecassus* (Evans) (Hymenoptera: Chrysidoidea) were used as an outgroup.

Sequences were aligned using CodonCode Aligner (v. 3.7.1.1, Codon Code Corporation). GBlocks [63] was used to delete highly divergent regions which could either not be unambiguously aligned or were saturated by multiple substitutions, or required assumption of multiple indels.

Preliminary analyses showed congruencies both in terms of topology and support between Bayesian and Maximum Likelihood phylogenetic analyses; we therefore decided to perform the analyses of this study using the faster Bayesian algorithm. Partitioned Bayesian inference searches were performed using MrBayes 3.1.2 [64] with the following 2 partitions: 18S rRNA gene and 28S rRNA gene. The best-fitting model of substitution for each partition was determined by AIC in jModeltest [65] and the GTR + I + G model was selected for both partitions.

To obtain a topology congruent with the most recent and well-established phylogenetic hypotheses, the following three constraints were used in the Bayesian phylogenetic analyses: (a) Sphecidae were constrained in basal position to the Crabronidae, Apidae and Megachilidae; (b) Crabronidae were constrained as monophyletic; (c) Apidae and Megachilidae were constrained as monophyletic [66].

We performed two runs of 10 million generations (started on random trees) and four incrementally heated Markov chains (using default heating values) each, sampling the Markov chains at intervals of 1000 generations. Stabilization and convergence of likelihood values was checked by visualizing the log likelihoods associated with the posterior distribution of trees in the program Tracer [67]. The first five million generations were conservatively discarded and five millions trees were retained post burn-in and summed to generate the majority rule consensus tree (Figure 1).

Likelihood unequivocal reconstruction of character evolution was performed using the ancestral state module implemented in MESQUITE (Version 2.75; [68]). The character evolution and ancestral states were reconstructed by mapping the character “food manipulation ability” (as a binary categorical trait, UtM vs. AtM) on the rooted topology generated from the previously described MrBayes analyses (Figure 1). As a major advantage, Maximum Likelihood takes branch lengths into account and allows quantifying the uncertainty associated with each reconstructed ancestral state [69]. For the likelihood apomorphic trends and ancestral state reconstruction we used the symmetrical Markov k-state one-parameter model (MK1) [70], which assumes a single rate of transition between two character states, and any particular change is equally probable. The likelihoods are reported as proportional likelihoods and are indicated as pie charts in Figure 1. Likelihood ratios at a node are compared by pairs, and the conventional cut-off point for assessing the significance of one state at a given node over the other (defined as a ‘rule-of-thumb’ [69]) is if their likelihoods differ by more than 2 log units (default setting in Mesquite).

### Statistical analyses

The relationships between flight morphology (FMR and WL) and food load manipulation ability (UtM vs. AtM) were analysed while accounting for common ancestry effects in the data (Felsenstein 1985; Garland et al. 1992). We controlled for phylogeny by means of phylogenetic generalized least-squares (PGLS) models [71-73], as implemented by the ‘ape’ library [74] of the software R (version 2.8.1) (R Development Core Team 2008). The phylogenetic variance–covariance matrix was obtained by the ‘corPagel’ function. The level of phylogenetic autocorrelation of species’ traits included in a PGLS model was expressed in terms of the λ index [72], that varies between 0 (phylogenetic independence) and 1 (species’ traits covary in proportion to their shared evolutionary history). PGLS models including the phylogenetic variance-covariance matrix multiplied by λ return phylogenetically corrected parameter estimates of covariation between phenotypic traits [72,73]. We tested whether the degree of phylogenetic dependence among traits was statistically significant by comparing a model where λ was set to 0 (i.e. assuming phylogenetic independence) with the model where it was allowed to reach its maximum likelihood value, according to a Brownian motion model of character evolution [74], by means of likelihood ratio (LR) tests [73]. We built two PGLS models, testing whether food load manipulation ability (0 = UtM; 1 = AtM) affected FMR and WL, respectively. In all models, we included body mass (log_10_-transformed) as an additional covariate. This was especially relevant for the model of WL, since this variable is known to scale allometrically with body mass across insect species according to a power function [51,75], and this was the case also in the present dataset (exponent of the power function = 0.394, F_1,22_ = 45.9, P < 0.001, r^2^ = 0.68). To linearize the relationship between WL and body mass, we therefore log_10_-transformed both variables before including them in all statistical models. Model residuals were normally distributed in all cases (Lilliefors test, P-values always > 0.16). Visual inspection of the data suggested that the variances in WL might differ according to food load manipulation (see Figure 2b). However, accounting for heterogeneity of variances in the PGLS model, by allowing the two levels of the covariate of food load manipulation to have different variances (see [76] for details), did not significantly improve model fit (LR test, P = 0.59), and did not qualitatively alter our conclusions (details not shown for brevity). We therefore report estimates from models not controlling for heteroscedasticity.

Finally, we analysed the covariation between FMR and WL by running a PGLS with WL as the dependent variable and FMR and body mass as predictors. Parameter estimates and mean values are reported together with their associated standard error (s.e.).

## Abbreviations

FMR: Flight muscle ratio; WL: Wing loading; AtM: Able to manipulate food load; UtM: Unable to manipulate food load; Mb: Body mass; Mt: Thorax mass; Loadmax: Maximum food load that females could theoretically carry in flight after a successful take-off; Mmax: Maximum total load (Load_max_ + M_b_) that females could theoretically carry in flight after a successful take-off; Aw: Total area of the wings; HW: Head width.

## Competing interests

The authors have declared that no competing interests exist.

## Authors’ contributions

CP designed the study. CP, LD and JS sampled the species in the field. CP and LD collected the morphological data. AC performed the phylogenetic reconstruction. DR, CP and NS carried out the statistical analyses. CP, DR and AC drafted the manuscript. All the authors read and approved the final manuscript.

## Supplementary Material

Additional file 1: Table S1 List of taxa and GenBank accession numbers of sequences used in the phylogenetic reconstruction. **Table S2**. Thorax mass (M_t_), maximum food load that females could theoretically carry in flight after a successful take-off (Load_max_), maximum total load that females could theoretically carry in flight (M_max_ = body mass + Load_max_), maximum % of food load that females could theoretically carry relative to body mass ((Load_max_/body mass) × 100)), total area of the wings (A_w_) and head width (HW) for the species used in the study. Category of food manipulation ability is reported (0 = unable to manipulate food load, 1 = able to manipulate food load). **Table S3**. PGLS models testing the effects of food load manipulation ability (0 = UtM; 1 = AtM) on flight muscle ratio (FMR) and (log_10_-transformed) wing-loading (WL) calculated based on dry body mass values, while controlling for head width (log_10_-transformed) as an index of body size (n = 21 species). The maximum likelihood estimate value of λ, assessing the degree of phylogenetic dependence among the tested variables (see Materials and methods), is shown for each model. Click here for file
