# Supplementary material for: Food load manipulation ability shapes flight morphology in females of central-place foraging Hymenoptera
Source: Front Zool. 2013 Jun 28;10:36. doi: 10.1186/1742-9994-10-36 (PMC3698194; doi:10.1186/1742-9994-10-36)
Supplement: Additional file 1: Table S1 — List of taxa and GenBank accession numbers of sequences used in the phylogenetic reconstruction. Table S2. Thorax mass (Mt), maximum food load that females could theoretically carry in flight after a successful take-off (Loadmax), maximum total load that females could theoretically carry in flight (Mmax = body mass + Loadmax), maximum % of food load that females could theoretically carry relative to body mass ((Loadmax/body mass) × 100)), total area of the wings (Aw) and head width (HW) for the species used in the study. Category of food manipulation ability is reported (0 = unable to manipulate food load, 1 = able to manipulate food load). Table S3. PGLS models testing the effects of food load manipulation ability (0 = UtM; 1 = AtM) on flight muscle ratio (FMR) and (log10-transformed) wing-loading (WL) calculated based on dry body mass values, while controlling for head width (log10-transformed) as an index of body size (n = 21 species). The maximum likelihood estimate value of λ, assessing the degree of phylogenetic dependence among the tested variables (see Materials and methods), is shown for each model. [file 1742-9994-10-36-S1.doc]

**Additional file 1**

Table S1. List of taxa and GenBank accession numbers of sequences used in the phylogenetic reconstruction.

| **Species used in the morphological analysis** | **Species used in the phylogenetic reconstruction** | **18S rRNA** | **28S rRNA** | **Source** |
| --- | --- | --- | --- | --- |
| *Amegilla dawsoni* | *Amegilla asserta* | GU244594 | GU244756 | Cardinal et al. 2010 |
| *Ammophila sabulosa* | *Ammophila sp.* JC134 | EF032321 | AF146672 | Schulmeister 2003, Carpenter & Wheeler 1999 |
| *Anthidium manicatum* | *Anthidium porterae* | GU244686 | GU244846 | Cardinal et al. 2010 |
| *Anthophora* sp. | *Anthophora montana* | AY995678 | AY654533 | Danforth et al. 2006a |
| *Apis mellifera* | *Apis mellifera* | HP516520 | HP510376 | Deng et al. (unpublished) |
| *Bembix olivacea* | *Bembix americana* | AY995580 | AY654459 | Danforth et al. 2006a |
| *Bembix sinuata* | *Bembix dentilabris* | AY995590 | AY654471 | Danforth et al. 2006a |
| *Bembix troglodytes* | *Bembix amoena* | - | EU367154 | Pilgrim et al. 2008 |
| *Bombus impatiens* | *Bombus diversus* | HM750223 | HM750236 | Cardinal et al. 2010 |
| *Bombus* sp. 1 | *Bombus ardens* | HM750224 | HM750237 | Cardinal et al. 2010 |
| *Bombus* sp. 2 | *Bombus mendax* | HM750222 | HM750235 | Cardinal et al. 2010 |
| *Eumenes* sp. 1 | *Eumenes fraternus* | EF190719 | EF190749 | Hines et al. 2007 |
| *Eumenes* sp. 2 | *Eumenes tripunctatus* | AF142514 | GU596725 | Carpenter and Wheeler 1999, Pickett & Carpenter 2010 |
| *Euodynerus* sp. | *Euodynerus megaera* | EF190723 | EF190753 | Hines et al. 2007 |
| *Megachile rotundata* | *Megachile pugnata* | AY995695 | AY654543 | Danforth et al. 2006a |
| *Monobia quandridens* | *Monobia quandridens* | MQU65154 | GU596750 | Pickett and Carpenter 2010, Whiting et al. 1997 |
| *Osmia rufa* | *Osmia lignaria* | GU244696 | GU244856 | Cardinal et al. 2010 |
| *Oxybelus* sp. | *Oxybelus abdominalis* | AY995584 | DQ072153 | Danforth et al. 2006a, 2006b |
| *Philanthus pulchellus* | *Philanthus gibbosus* | AY995585 | AY654464 | Danforth et al. 2006a |
| *Philanthus triangulum* | *Philanthus sp.* CSM-2006 | DQ353551 | DQ353559 | Moreau et al. 2006 |
| *Polistes dominulus* | *Polistes metricus* | EF190710 | GU596782 | Hines et al. 2007, Pickett and Carpenter 2010 |
| *Sceliphron curvatum* | *Sceliphron caementarium* | AY995593 | AY654468 | Danforth et al. 2006a |
| *Sceliphron destillatorium* | *Sceliphron laetum* | - | JF510019 | Hoggard et al. 2011 |
| *Sphex rufocinctus* | *Sphex lucae* | AY995592 | AY654466 | Danforth et al. 2006a |
| *Vespula germanica* | *Vespula germanica* | AY919030 | GU596815 | Castro and Mark unpublished, Pickett and Carpenter 2010 |
| *Vespula maculifrons* | *Vespula maculifrons* | EF190708 | EF190738 | Hines et al. 2007 |
| *Vespula vulgaris* | *Vespula squamosa* | EF190730 | GU596817 | Hines et al. 2007, Pickett and Carpenter 2010 |
| *Xylocopa varipuncta* | *Xylocopa pubescens* | GU244748 | GU244908 | Cardinal et al. 2010 |
| *-* | *Scolebythus madecassus* (outgroup) | GQ410609 | GQ374716 | Heraty et al. 2011 |

**References**

Cardinal, S., J. Straka, and B. N. Danforth. 2010. Comprehensive phylogeny of apid bees reveals the evolutionary origins and antiquity of cleptoparasitism. Proc. Nat. Acad. Sci. U. S. A. 107:16207-16211.

Carpenter, J. M., and W. C. Wheeler. 1999. Towards simultaneous analysis of morphological and molecular data in Hymenoptera. Zool. Scr. 28:251-260.

Danforth, B. N., S. Sipes, J. Fang, and S. G. Brady. 2006a. The history of early bee diversification based on five genes plus morphology. Proc. Nat. Acad. Sci. U. S. A. 103:15118-15123.

Danforth, B. N., J. Fang, and S. D. Sipes. 2006b. Analysis of family-level relationships in bees (Hymenoptera: Apiformes) using 28S and two previously unexplored nuclear genes: CAD and RNA polymerase II. Mol. Phylogenet. Evol. 39:358-372.

Heraty, J., F. Ronquist, J. M. Carpenter, D. Hawks, S. Schulmeister, A. P. Dowling, D. Murray, J. Munro, W. C. Wheeler, N. Schiff, and M. Sharkey. 2011. Evolution of the hymenopteran megaradiation. Mol. Phylogenet. Evol. 60:73-88.

**Hines, H. M.**, J. H. Hunt, T. K. O'Connor, J. J. Gillespie, and S. A. Cameron. 2007. Multigene phylogeny reveals eusociality evolved twice in vespid wasps. Proc. Natl. Acad. Sci. U. S. A, 104:3295-3299.

Hoggard, S. J., P. D. Wilson, A. J. Beattie, and A. J. Stow. 2011. Social complexity and nesting habits are factors in the evolution of antimicrobial defences in wasps. PLoS ONE 6(7): e21763. doi:10.1371/journal.pone.0021763.

Moreau, C. S., C. D. Bell, R. Vila, S. B. Archibald, and N. E. Pierce. 2006. Phylogeny of the ants: Diversification in the age of angiosperms. Science

312:101-104.

Pickett, K. M., and J. M. Carpenter. 2010. Simultaneous analysis and the origin of eusociality in the Vespidae (Insecta: Hymenoptera) Arth. Syst. Phyl. 68:3-33.

Pilgrim, E. M., C. D. von Dohlen, and J. P. Pitts. 2008. Molecular phylogenetics of Vespoidea indicate paraphyly of the superfamily and novel relationships of its component families and subfamilies. Zool. Scr., 37:539-560.

Schulmeister, S. 2003. Simultaneous analysis of basal Hymenoptera (Insecta) introducing robust-choice sensitivity analysis. Biol. J. Linn. Soc. 79:245-275.

Whiting, M. F., J. C. Carpenter, Q. D. Wheeler, and W. C. Wheeler. 1997. The Strepsiptera problem: phylogeny of the holometabolous insect orders inferred from 18S and 28S ribosomal DNA sequences and morphology. Syst. Biol. 46:1-68.

Table S2. Thorax mass (M_t_), maximum food load that females could theoretically carry in flight after a successful take-off (Load_max_), maximum total load that females could theoretically carry in flight (M_max_ = body mass+Load_max_), maximum % of food load that females could theoretically carry relative to body mass ((Load_max_/body mass) × 100)), total area of the wings (A_w_) and head width (HW) for the species used in the study. Category of food manipulation ability is reported (0 = unable to manipulate food load, 1 = able to manipulate food load).

| **Taxonomy** | **Species** | **Food manipulation ability** | **M_t_ (g)** | **Load_max_ (g)** | **% Load_max_** | **M_max_ (g)** | **A_w_ (cm^2^)** | **HW (mm)** |
| --- | --- | --- | --- | --- | --- | --- | --- | --- |
| Apoidea: Apidae | *Amegilla dawsoni* | 1 | - | - | - | - | - | - |
| Apoidea: Apidae | *Anthophora* sp. | 1 | 0.055 | 0.146 | 109.77 | 0.279 | 0.730 | 3.60 |
| Apoidea: Apidae | *Apis mellifera* | 1 | 0.035 | 0.081 | 86.17 | 0.175 | 0.560 | 3.82 |
| Apoidea: Apidae | *Bombus impatiens* | 1 | - | 0.076 | 37.81 | 0.277 | 0.720 | - |
| Apoidea: Apidae | *Bombus* sp. 1 | 1 | 0.080 | 0.200 | 96.15 | 0.408 | 0.930 | 4.26 |
| Apoidea: Apidae | *Bombus* sp. 2 | 1 | 0.086 | 0.231 | 113.23 | 0.435 | 1.04 | 4.34 |
| Apoidea: Apidae | *Xylocopa varipuncta* | 1 | - | 0.713 | 85.08 | 1.551 | 2.53 | - |
| Apoidea: Megachilidae | *Anthidium manicatum* | 1 | 0.056 | 0.128 | 83.12 | 0.282 | 0.737 | 5.30 |
| Apoidea: Megachilidae | *Megachile rotundata* | 1 | 0.034 | 0.071 | 69.61 | 0.173 | 0.580 | 4.10 |
| Apoidea: Megachilidae | *Osmia rufa* | 1 | 0.069 | 0.165 | 88.23 | 0.352 | 0.83 | 4.80 |
| Apoidea: Sphecidae | *Ammophila sabulosa* | 0 | 0.011 | 0.03 | 115.38 | 0.056 | 0.35 | 2.52 |
| Apoidea: Sphecidae | *Sceliphron curvatum* | 0 | 0.040 | 0.119 | 143.37 | 0.202 | 0.88 | 4.00 |
| Apoidea: Sphecidae | *Sceliphron destillatorium* | 0 | 0.086 | 0.247 | 136.46 | 0.428 | 1.52 | 4.78 |
| Apoidea: Sphecidae | *Sphex rufocinctus* | 0 | 0.053 | 0.151 | 127.96 | 0.269 | 1.053 | 4.30 |
| Apoidea: Crabronidae | *Bembix olivacea* | 0 | 0.052 | 0.156 | 143.12 | 0.265 | 0.8 | 4.03 |
| Apoidea: Crabronidae | *Bembix sinuata* | 0 | 0.075 | 0.226 | 143.04 | 0.384 | 0.8 | 5.21 |
| Apoidea: Crabronidae | *Bembix troglodytes* | 0 | - | - | - | - | - | - |
| Apoidea: Crabronidae | *Oxybelus* sp. | 0 | 0.003 | 0.007 | 87.5 | 0.015 | 0.086 | 1.91 |
| Apoidea: Crabronidae | *Philanthus pulchellus* | 0 | 0.017 | 0.044 | 102.32 | 0.087 | 0.52 | 3.55 |
| Apoidea: Crabronidae | *Philanthus triangulum* | 0 | 0.039 | 0.102 | 110.87 | 0.194 | 0.85 | 4.59 |
| Vespoidea: Vespidae | *Polistes dominulus* | 1 | 0.025 | 0.061 | 93.85 | 0.126 | 0.77 | 3.30 |
| Vespoidea: Vespidae | *Vespula germanica* | 1 | - | - | - | - | - | - |
| Vespoidea: Vespidae | *Vespula maculifrons* | 1 | - | - | - | - | - | - |
| Vespoidea: Vespidae | *Vespula vulgaris* | 1 | 0.028 | 0.063 | 80.77 | 0.141 | 0.57 | 3.68 |
| Vespoidea: Vespidae | *Eumenes* sp. 1 | 0 | 0.021 | 0.053 | 110.42 | 0.101 | 0.61 | 3.14 |
| Vespoidea: Vespidae | *Eumenes* sp. 2 | 0 | 0.017 | 0.043 | 102.38 | 0.085 | 0.39 | 3.72 |
| Vespoidea: Vespidae | *Euodynerus* sp. | 0 | 0.019 | 0.045 | 91.84 | 0.094 | 0.73 | 3.24 |
| Vespoidea: Vespidae | *Monobia quandridens* | 0 | - | - | - | - | - | - |

Table S3. PGLS models testing the effects of food load manipulation ability (0 = UtM; 1 = AtM) on flight muscle ratio (FMR) and (log_10_-transformed) wing-loading (WL) calculated based on dry body mass values, while controlling for head width (log_10_-transformed) as an index of body size (n = 21 species). The maximum likelihood estimate value of λ, assessing the degree of phylogenetic dependence among the tested variables (see Methods), is shown for each model.

| **Model** | **Estimate (s.e.)** | **t** | **P** | **λ** |
| --- | --- | --- | --- | --- |
| *FMR* | | | | |
| Food load manipulation ability | -0.061 (0.016) | -3.75 | 0.002 | 0.11 |
| Head width | 0.037 (0.072) | 0.52 | 0.61 |  |
|  | | | | |
| *WL* | | | | |
| Food load manipulation ability | 0.286 (0.110) | 2.58 | 0.019 | 1.00 |
| Head width | 0.706 (0.230) | 3.07 | 0.007 |  |
